# An external validation of coding for childhood maltreatment in routinely collected primary and secondary care data

**DOI:** 10.1038/s41598-023-34011-3

**Published:** 2023-05-19

**Authors:** Ann John, Joanna McGregor, Amanda Marchant, Marcos DelPozo-Baños, Ian Farr, Ulugbek Nurmatov, Alison Kemp, Aideen Naughton

**Affiliations:** 1 Population Data Science, Data Science Building, Swansea University Medical School, Swansea University, Singleton Park, Swansea, SA2 8PP UK; 2 School of Medicine, Cardiff University, Neuadd Meirionnydd, Cardiff, CF14 4YS UK; 3 Public Health Wales NHS Trust, Cardiff, CF10 4BZ UK

**Keywords:** Human behaviour, Health care, Medical research, Risk factors

## Abstract

Validated methods of identifying childhood maltreatment (CM) in primary and secondary care data are needed. We aimed to create the first externally validated algorithm for identifying maltreatment using routinely collected healthcare data. Comprehensive code lists were created for use within GP and hospital admissions datasets in the SAIL Databank at Swansea University working with safeguarding clinicians and academics. These code lists build on and refine those previously published to include an exhaustive set of codes. Sensitivity, specificity and positive predictive value of previously published lists and the new algorithm were estimated against a clinically assessed cohort of CM cases from a child protection service secondary care-based setting—‘the gold standard’. We conducted sensitivity analyses to examine the utility of wider codes indicating Possible CM. Trends over time from 2004 to 2020 were calculated using Poisson regression modelling. Our algorithm outperformed previously published lists identifying 43–72% of cases in primary care with a specificity ≥ 85%. Sensitivity of algorithms for identifying maltreatment in hospital admissions data was lower identifying between 9 and 28% of cases with high specificity (> 96%). Manual searching of records for those cases identified by the external dataset but not recorded in primary care suggest that this code list is exhaustive. Exploration of missed cases shows that hospital admissions data is often focused on the injury being treated rather than recording the presence of maltreatment. The absence of child protection or social care codes in hospital admissions data poses a limitation for identifying maltreatment in admissions data. Linking across GP and hospital admissions maximises the number of cases of maltreatment that can be accurately identified. Incidence of maltreatment in primary care using these code lists has increased over time. The updated algorithm has improved our ability to detect CM in routinely collected healthcare data. It is important to recognize the limitations of identifying maltreatment in individual healthcare datasets. The inclusion of child protection codes in primary care data makes this an important setting for identifying CM, whereas hospital admissions data is often focused on injuries with CM codes often absent. Implications and utility of algorithms for future research are discussed.

## Introduction

Childhood Maltreatment (CM) is a serious global public health concern and is associated with long-lasting psychological and physical adverse health outcomes^1^. The World Health Organisation (WHO) in collaboration with the International Society for the Prevention of Child Abuse and Neglect (ISPCAN) recommend the development of clear operational case definitions and common reliable methods of identification. They have defined CM as "all forms of physical and/or emotional ill-treatment, sexual abuse, neglect or negligent treatment or commercial or other exploitation, resulting in actual or potential harm to the child's health, survival, development or dignity in the context of a relationship of responsibility, trust or power"^2^.

Estimates suggest 20–25% of the adult population may have experienced some form of maltreatment during childhood^3–5^. Official statistics from child protection agencies and authorities underestimate the true incidence and prevalence in society, as unrecognised and unreported cases would be absent^6^. The Office of National Statistics (ONS), as at 31 March 2019, 52,260 children in England (43 cases per 10,000) were subject to a Child Protection Plan and a further 2,820 children in Wales (45 cases per 10,000) were on the Child protection register^5^. The Crime Survey for England and Wales (CSEW) conducted in 2019 estimated that 1 in 5 adults experienced child abuse before the age of 16 years^7^.

Health professionals, such as general practitioners (GPs), in regular and long-standing contact with children and their families are well placed to identify children ‘at risk’^8^. The National Institute for Health and Clinical Excellence (NICE) and the General Medical Council (GMC) highlight the importance of the role of health professionals in identifying and documenting concerns of ‘suspected’ CM^9,10^ as victims may be reluctant to confide or even be unaware that they are being maltreated.

The availability of population-based routinely collected data such as medical records from primary and secondary care sources have the potential to be a rich data resource for research on CM. Hospital data has been used for CM surveillance in the UK^11^ and abroad^12–15^. GP data has a wider range of maltreatment-related concerns due to the extensive code nomenclature. While previous research with routinely collected data has explored CM, there is yet to be an agreed validated list of codes for its identification.

There have been several attempts to identify maltreatment and maltreatment related concerns in primary care. These wider code lists have been designed to capture clinical concern relating to suspected or possible maltreatment^16^. These include codes such as ‘referral to social services.’ Such codes have been selected to reflect a threshold that should trigger further action by health professionals^17^. While these codes may indicate potential vulnerability, these codes do not necessarily indicate maltreatment. Someone may be referred to social services for a variety of other reasons (e.g., parental illness or disability). A validation exercise of these codes in three practices indicated high specificity for ‘considered maltreatment’, however considerable under-reporting of CM was found compared with community studies^17^.

Other code lists for CM have been developed and include categorization into incident and prevalent code lists (with the latter including codes indicative of historical maltreatment^18^). However, these code lists did not include codes for maltreatment-related concerns and found a considerably lower incidence and prevalence than previous research encompassing a broader list of codes^19^. A recent paper set out to identify and validate adverse childhood experiences (ACEs), including CM, in routinely collected healthcare data^20^. This project developed and statistically validated lists using both maternal and child health records from two years before birth to five years after. This study aimed to identify indicators that reflected a continuum of clinically meaningful risk groups consistent with previous ACE definitions. This list represents one of the most comprehensive in the current literature. Incidence and prevalence using this list has not yet been explored and it has yet to be externally validated.

Standardised, reliable, and well-validated methods of identifying CM are needed to better understand the true extent and consequences of CM. In this study we aim to build on the current work. We will assess the ability to identify cases of CM in primary care and hospital admissions data using an externally validated secondary care-based clinically-assessed CM cohort as part of a child protection service as the gold-standard following the methodology of previous validation exercises^21^. We will explore recording of CM in individual healthcare datasets and discuss the strengths and limitations of coding in each setting, and explore the utility of an algorithm combining these two data sources. We will explore the reasons for missed and incorrectly identified cases in each healthcare setting. We also assess variation in CM rates over time.

## Methods

### Study design

This is a retrospective e-cohort study.

### Ethical approval

Approval was granted on 4/12/2018 by the Swansea University Information Governance Review Panel (IGRP) (approval number 0809), an independent body consisting of a range of government, regulatory and professional agencies (British Medical Association (BMA), National Research Ethics Service (NRES), Involving People, NHS Wales Informatics Service and Public Health Wales (PHW) NHS Trust) and members of the public, which grants approval to studies conducted within the SAIL Databank. All methods were performed in accordance with the relevant guidelines and regulations and in line with the permissions granted under these ethical approvals. All data within the SAIL gateway are treated in accordance with the Data Protection Act 2017 and are compliant with the General Data Protection Regulation (GDPR).

Informed consent was not required as this study utilizes fully anonymised data in accordance with the GDPR.

### Data source

We linked data on an individual level via the Adolescent Mental Health Data Platform (ADP), an international data platform that supports mental health research in children and young people (CYP). For our study, the ADP used datasets from the SAIL Databank, a repository of routinely collected health and education datasets for the population of Wales^22,23^. All data are treated in accordance with the Data Protection Act 2018. The following datasets were linked a patient level:

Welsh Demographic Service (WDS), Welsh Index of Multiple Deprivation containing deprivation scores for all lower super output areas in Wales; GP database (GPD), containing information for all GP interactions covering 79% of the Welsh population; Patient Episode Database for Wales (PEDW), containing data for all NHS Wales hospital admissions.

### Externally validated dataset

Cardiff and Vale University Health Board Minimum dataset for CM (CVCM) was imported into SAIL databank via the split-file method for anonymisation. The CVCM dataset comprises 3622 clinical assessments pertaining to 3123 children for suspected CM and includes date of assessment, type of abuse suspected (i.e., physical, neglect, sexual), reason for suspicions, details, and confirmation of findings. Three quarters (75.8%, 2747/3622) of the assessments were conducted on the basis of suspicion of physical abuse, 17% were sexual abuse and around 4% were neglect. For the purpose of this study CM was examined overall with no further stratification by maltreatment type due to the number of cases available within each sub-category. Within the dataset, the outcomes of the clinical assessments were divided into three categories—confirmed maltreatment, possible maltreatment and no maltreatment. Of the 3,123 children, 388 (12.4%) had been seen on more than one occasion and 2889/3123 (92.5%) were under 18, living in Wales and were assessed between 2004 and 2018.

### Study population

Individuals aged 0–17 registered with a SAIL-supplying GP from 01.01.2004–10.10.2020 were selected as the baseline population. Data collection began at GP registration date plus six months if newly registered (to avoid misclassification due to retrospective recording at registration), except for the under 1 s, who were followed from GP registration date or study onset whichever was the latest. Data collection ended on the date of GP de-registration, death, 18th birthday or study end whichever was the sooner. Individuals could supply multiple data periods.

Two study cohorts were created for the purpose of this study. The first for the validation exercise, the second for exploring incidence and prevalence (Supplementary File 1 Fig. S1).

#### Validation cohort

For validation purposes the CVCM dataset was linked with routinely collected data in the SAIL databank following the criteria above at the level of the individual.

We required one assessment in the CVCM dataset per child. Cases were divided into confirmed CM and not confirmed CM (the latter encompassing no maltreatment and possible cases) based on clinician assessment. Therefore, for the children seen on more than one occasion, a hierarchical rule system was adopted, whereby we used the most recent assessment date of any case that had ever been assessed as ‘confirmed CM’, followed by the most recent assessment date for the ‘no CM’ cases.

Only children within the CVCM dataset who were registered to a SAIL-supplying GP, supplied a minimum of 6 months data including the index date, were included for comparisons to the routine data.

#### Incidence and prevalence cohort

Individuals registered with a SAIL-supplying GP following the inclusion criteria above (independent of linkage to the CVCM dataset) were included in the incidence and prevalence analysis. Data collection for each year began on the 1st on January or the start of follow-up as defined above, whichever was the later, and ended on the 31st December or the end of data collection whichever was the sooner. Person time was calculated between the start and end dates for each year.

### Measures

Age and deprivation indices were collected based on the onset of data collection for each year. Individuals were stratified by sex, age group (< 1 y, 1–4 y, 5–9 y, 10–14 y, 15–17 to align with data from the National Public Health Service for Wales) and quintiles of deprivation.

Maltreatment was identified from GP data using primary care read codes and from hospital admissions data using ICD 10 codes. The development of the code lists is described below. Code lists for primary care and hospital admissions were developed to map onto one another as closely as possible. However, the coding nomenclature in primary care is broader and encompasses codes not available in hospital admissions data (e.g., child protection codes).

Incidence and prevalence of CM over time was examined in primary care data and admissions data. Incidence was defined as no record in the previous 12 months. Prevalence was defined as any record of maltreatment within a given year, independent of any previous events^21,24^.

### Development of code lists to identify CM

In order to fully explore the coding of CM in individual healthcare settings two sets of code lists were developed: Read codes for use in GP data and ICD-10 codes for admissions data. While we mapped these two sets of code lists as closely to one another as possible, these distinct coding systems contain different levels of detail. GP data contains a broader coding nomenclature than admissions data, including communication from other healthcare settings and child protection agencies. The utility of each set of codes will be assessed both separately and in combination.

Our codes lists were developed from existing literature^15,16,19,20,25–28^, conducting our own searches for codes that may be indicate maltreatment, risk, or cause for concern; and finally based upon clinician judgement. In keeping with previous research, we tiered our code list into those strongly indicating CM/confirmed maltreatment, referred to here as ‘Confirmed CM’ codes, and those codes that may indicate possible or suspected maltreatment or potential vulnerability^16,20^, referred to here as ‘’Possible CM’ codes. For the Confirmed CM codes in primary care we further divided into prevalent codes and incident codes with codes indicating historical maltreatment (e.g. history of child abuse) excluded from the incident list^18^.

Additional sensitivity analysis was conducted to refine previous lists testing sensitivity and specificity of different subgroups and using clinical input to determine whether these codes are appropriate (excluding for example codes such as ‘parents on benefits’ and ‘self-neglect.’ Supplementary analysis available on request).

### Confirmed CM

Within the category of Confirmed CM were terms that unequivocally stated the existence of maltreatment. This included maltreatment syndromes, history/victim of abuse, prostitution, genital mutilation and criminal neglect/abandonment of baby and child protection categories. Child protection is a response to confirmed maltreatment that has already taken place. This is distinct from safeguarding which refers to measures put in place to prevent harm. Child protection and the presence of maltreatment would be determined by a case conference and this information communicated to GPs and as such should appear in primary care data. However, based on conversations with clinical colleagues child protection information may not always be present in the CVCM dataset and are rarely available in hospital admissions data.

### Possible CM

Within the category of ‘Possible CM’ are codes that may indicate risk and vulnerability of children that may co-occur with maltreatment. However, they are not sufficient to indicate maltreatment in isolation. These codes fell into six categories:

1. At risk/safeguarding codes: For example, ‘at risk of abuse’ or ‘safeguarding example’. Safeguarding was distinguished from child protection which are included in the Confirmed CM list (see above). A child or young person is a safeguarding concern when they are living in circumstances where there is a significant risk of abuse (physical, sexual, emotional or neglect). At-risk codes may not be specific enough to record an event of Confirmed CM, however these codes have utility for identifying children at significant risk. This may have implications for long-term outcomes and future research with vulnerable children. There is no equivalent of these codes in hospital admissions data, so these were searched in GP data only.
2. Other social care: For example, ‘referred to social worker’ or ‘in foster care.’ Social care codes not specifically related to child-protection may also be useful for identifying potentially vulnerable children. However, there are many reasons why a child may have contact with social care that may not be related to maltreatment (e.g., parent or child disability and mental or physical health problems of either the parent or the child). There is no equivalent of these codes in hospital admissions data, so these were searched in GP data only.
3. Family circumstances: This includes codes ‘child abuse in family’ or ‘family member on protection register’. These codes are indicative of risk but do not necessarily indicate CM for the patient in question
4. Alleged/suspected maltreatment: For example, ‘suspected child abuse’, ‘alleged abuse’
5. Rib/limb fractures: For example, ‘multiple rib fractures’
6. Assaults: This included general codes such as ‘Assault’ and more specific codes such as ‘physical assault at home’.

The full lists of codes can be found in Supplementary Files 2–5.

### Statistical analysis

#### Validation exercise

We compared cases of CM identified in the routinely collected datasets to the clinical assessment outcomes recorded in the externally validated CVCM cohort to establish levels of agreement. We utilised the ‘Confirmed CM’ and all ‘Possible CM’ code lists applied to both the 12 months either side of the recorded maltreatment/suspected maltreatment event in the CVCM dataset and searching at any time during the follow-up period. This may indicate the algorithms’ ability to identify individual events compared with utility in identifying maltreated individuals. We further utilised the ‘Confirmed CM’ list adding categories of ‘Possible CM’ codes (e.g. ‘Confirmed CM/other social care’, ‘Confirmed CM/suspected or alleged’). We calculated sensitivity, specificity, and positive predictive values (PPV), negative predictive values (NPV) and 95% confidence intervals and explored reasons for identifying false negatives and false positives. Prevalence of maltreatment was reported because this number affects the PPV and NPV. However, this was the prevalence only within the narrowly defined study population, which was defined by hospital evaluation protocols. It is not the prevalence within the general population of the hospital or community^29^.

#### Trends over time

To assess variation over time, we calculated change in annual incidence and 95% CIs for Confirmed CM and Possible CM rates per 1000 PYAR at risk between 01.01.2004 and 10.10.2020 for children aged under 18 years of age. Poisson regression was undertaken to investigate the adjusted association between incidence and prevalence of maltreatment, and year of diagnosis, sex, age group and deprivation. The significance of the variables in the Poisson regression modelling were assessed using Wald tests. Confidence intervals (CIs) for rates were estimated using two-tailed mid-p exact CIs (assuming Poisson distribution)^30^. Statistical analyses were conducted using SPSS statistical software (version 22).

## Results

### Study population

The study population comprised 1,078,486 CYP (aged 0–17 years), registered to a SAIL-supplying general practice with at least six months’ worth of GP data between 01.01.2004 and 10.10.2020. They contributed 7,270,724 person years’ worth of data. The mean follow-up for each individual was six years.

There were 2205 CYP who had been clinically assessed for CM out of the population (n = 1,078,486), which comprised the maltreatment validation e-cohort. Of these, around a quarter (25.1%) were confirmed maltreatment. Maltreatment was not confirmed for the remaining 1652 (74.9%) cases (encompassing both possible and no maltreatment categories).

### Validation exercise 1: primary care data

#### Validation results 1a: previously published lists

The sensitivity, specificity and positive predictive values of previously published code lists relating to primary care are shown in Table 1.

**Table 1:** Sensitivity (95% CI), Specificity (95% CI), PPV(95% CI) NPV(95% CI) of previously published lists when run through validation exercise against gold standard.

		Sensitivity		Specificity		PPV^a^		NPV^a^	
		Time 1^b^	Time 2^c^	Time 1^b^	Time 2^c^	Time 1^b^	Time 2^c^	Time 1^a^	Time 2^b^
Chandan et al.^18^	Confirmed CM incidence^d^	7.6 (5.6–10.2)	9.9 (7.6–12.8)	98.9 (98.2–99.3)	97.9 (97.0–98.5)	70.0 (56.6–80.8)	61.1 (50.2–71.0)	76.2 (74.3–78.0)	76.5 (74.6–78.2)
	Confirmed CM prevalence^e^	8.3 (6.2–11.0)	11.6 (9.1–14.6)	98.5 (97.7–99.0)	97.4 (96.5–98.1)	64.8 (52.5–75.5)	59.8 (49.9–69.0)	76.2 (74.4–78.0)	76.7 (74.8–78.5)
Chandan et al.^28^	Confirmed CM^f^ + maltreatment related	51.0 (46.7–55.2)	64.4 (60.2–68.3)	90.1 (88.6–91.5)	86.1 (84.4–87.7)	63.4 (58.7–67.8)	60.9 (56.8–64.8)	84.6 (82.8–86.2)	87.8 (86.1–89.4)
Syed et al.^20^	Confirmed CM^f^	57.9 (53.6–62.0)	68.0 (63.9–71.8)	89.5 (87.9–90.9)	85.8 (84.0–87.4)	64.8 (60.4–69.0)	61.5 (57.5–65.4)	86.4 (84.6–88.0)	88.9 (87.2–90.4)
	Confirmed CM^f^ + other social care codes	48.6 (44.4–52.9)	59.1 (54.9–63.2)	91.1 (89.6–92.4)	87.1 (85.4–88.7)	64.7 (59.8–69.2)	60.6 (56.3–64.7)	84.1 (82.3–85.8)	86.4 (84.7–88.0)
	Confirmed CM^f^ + suspected maltreatment	59.1 (54.9–63.2)	69.3 (65.2–73.0)	89.1 (87.5–90.5)	85.7 (83.8–87.2)	64.5 (60.1–68.6)	61.7 (57.7–65.5)	86.7 (85.0–88.3)	89.3 (87.6–90.7)

^a^PPV and NPV based on prevalence in validation population.

^b^Codes present in GP record within 12 months before or after index date.

^c^Codes present in GP record at any time.

^d^Incident case refers to a new maltreatment event (either first in records or no record within previous 12 months). Codes related to historical maltreatment are excluded.

^e^Prevalent case refers to an event independent of whether there has been a previous maltreatment date. All codes including those related to historical maltreatment are included.

^f^Confirmed CM here refers to prevalence list with historical codes included.

Previously published code lists from^18^ performed with excellent specificity (> 97%). However, sensitivity was low ranging from 7.6% for incident code lists explored in the 12 months either side of an index date to 11.6% for prevalent code lists where records were searched at any time. While specificity for these lists is high around 90% of cases would be missed.

Sensitivity is improved in codes lists from^16^ ranging from 51.0 to 64.4%. However, specificity is lower ranging from 86.1 to 90.1%. This list included a wider range of maltreatment related codes including codes related to child protection procedures. While sensitivity is higher, this wider code list allows a wider range of potentially indicative codes.

The highest performing previously published code list was that published by^20^ (Table 1). To make this list comparable with the updated algorithm we ran three versions of this list through the validation exercise. Sensitivity ranged from 48.6%-69.3% and specificity from 85.5 to 91.1% and depending on time restrictions and subset of list used. Inclusion of codes related to wider social care outside of child protection increased sensitivity with a negative impact on specificity. Utilising codes categorised as maltreatment and suspected maltreatment resulted in the highest sensitivity ranging from 59.1 to 69.3 with the lowest specificity of 85.7–89.1.

#### Validation results 1b: current code list

Results of the validation exercise with the current code list are shown in Table 2. This includes sensitivity analysis accounting for the impact of adding or removing various groups of codes.

**Table 2:** Sensitivity (95% CI), Specificity(95% CI), PPV(95% CI) NPV(95% CI) of code lists for child maltreatment included sensitivity analysis of additional and exclusion of categories of indicative/at-risk codes in primary care.

	Sensitivity		Specificity		PPV^a^		NPV^a^	
	Time 1^b^	Time 2^c^	Time 1^b^	Time 2^c^	Time 1^b^	Time 2^c^	Time 1^b^	Time 2^c^
Confirmed CM^d^ prevalence^e^	43.0 (38.9–47.3)	54.1 (49.8–58.3)	91.3 (89.9–92.6)	87.7 (86.0–89.2)	82.7 (80.9–84.4)	85.1 (83.3–86.7)	82.7 (80.9–84.4)	85.1 (83.3–86.7)
Confirmed CM^d^ incidence^f^	39.4 (35.3–43.6)	51.4 (47.1–55.6)	92.0 (90.6–93.2)	88.1 (86.5–89.6)	62.3 (57.0–67.3)	59.2 (54.6–63.6)	81.9 (80.1–83.7)	84.4 (82.6–86.1)
Confirmed CM^g^ + ‘child protection’ codes removed	19.2 (16.0–22.8)	31.1 (27.3–35.2)	96.4 (95.4–97.2)	93.4 (92.1–94.5)	64.2 (56.4–71.4)	61.2 (55.2–66.9)	78.1 (76.2–79.9)	80.2 (78.3–81.9)
Confirmed CM^g^ + ‘at risk'	44.8 (40.7–49.1)	55.5 (51.3–59.7)	91.1 (89.6–92.4)	87.4 (85.7–89.0)	62.8 (57.8–67.5)	59.6 (55.2–63.9)	83.1 (81.3–84.8)	85.4 (83.7–87.1)
Confirmed CM^g^ + ‘family circumstances'	45.0 (40.8–49.3)	58.2 (54.0–62.4)	91.0 (89.5–92.3)	86.8 (85.1–88.4)	62.6 (57.6–67.3)	59.6 (55.3–63.8)	83.2 (81.4–84.9)	86.1 (84.4–87.7)
Confirmed CM^g^ + ‘other social care^h^’	54.6 (50.4–58.8)	66.4 (62.2–70.3)	89.5 (87.9–90.9)	86.0 (84.2–87.6)	63.4 (58.9–67.7)	61.3 (57.2–65.2)	85.5 (83.7–87.1)	88.4 (86.7–89.9)
Confirmed CM^g^ + ‘suspected/alleged maltreatment’	43.2 (39.1–47.5)	54.2 (50.0–58.4)	91.3 (89.9–92.6)	87.7 (86.0–89.2)	62.6 (57.5–67.4)	59.6 (55.2–63.9)	82.8 (80.9–84.5)	85.1 (83.3–86.8)
Confirmed CM^g^ + ‘rib/limb fractures’	43.4 (39.2–47.7)	54.4 (50.2–58.6)	91.3 (89.9–92.6)	87.7 (86.0–89.2)	62.7 (57.6–67.5)	59.7 (55.3–64.0)	82.8 (81.0–84.5)	85.2 (83.4–86.8)
Confirmed CM^g^ + ‘assaults’	48.6 (44.4–52.9)	58.8 (54.5–62.9)	91.2 (89.7–92.5)	87.4 (85.7–89.0)	25.1 (23.3–27.0)	25.1 (23.3–27.0)	65.0 (60.1–69.5)	61.0 (56.7–65.1)
Confirmed CM^g^ + all possible CM codes	61.1 (56.9–65.2)	71.8 (67.8–75.5)	89.0 (87.3–90.4)	85.0 (83.2–86.7)	25.1 (23.3–27.0)	25.1 (23.3–27.0)	65.0 (60.7–69.1)	61.6 (57.7–65.3)

^a^PPV and NPV based on prevalence in validation population.

^b^Codes present in GP record within 12 months before or after index date.

^c^Codes present in GP record at any time.

^d^Confirmed CM codes encompass maltreatment related syndromes, child protection codes, genital mutilation, and prostitution.

^e^Prevalent case refers to an event independent of whether there has been a previous maltreatment date. All codes including those related to historical maltreatment are included.

^f^Incident case refers to a new maltreatment event (either first in records or no record within previous 12 months). Codes related to historical maltreatment are excluded.

^g^Confirmed CM here refers to the prevalence list with historical codes included.

^h^‘Other social care’ refers to social care codes that do not specific child protection for example ‘child in care’ or ‘social services involved’.

When ‘child protection’ codes are removed sensitivity drops markedly from 43.0% (95% CI 38.9–47.3) to 19.2% (95% CI 16.0–22.8) at 12 months or 54.1%(95% CI 47.1–55.6) to 31.1(95% CI 27.3–35.2) at any time. Specificity is improved slightly from 91.4% (95% CI 89.9–92.7) to 96.4%(95% CI 95.4–97.2) at 12 months and 88.1%(95% CI 86.5–89.6) to 93.4%(95% CI 92.1–89.6) at any time.

Sensitivity is improved with each group of codes added with ‘Other Social Care’ codes having the biggest impact on sensitivity (sensitivity at 12 months either side 54.6% [95% CI 50.4–58.8] at any time 66.4%[95% CI 62.2–70.3]). However, these also have a detrimental impact on specificity (12 months 89.65% [95% CI 87.9–90.9] at any time 86.0% [95% CI 84.2–87.6]). Assault codes also have a large impact on the algorithm increasing sensitivity to 48.6% (95% CI 44.4–52.9) in the 12 months either side of an index date and to 58.8%(95% CI 54.5–62.9) at any time. Inclusion of these codes has a small impact on specificity (12 months 91.2% [95% CI 89.7–92.5]; at any time 87.4%[95% CI 85.7–89.0]). The most frequently used ‘assault’ code was ‘[X]Assault’. Refining these codes to only include ‘assaults occurring at home’ did not impact the algorithm as they were rarely used.

When all additional codes are added (‘at risk’, ‘family circumstances’, ‘other social care’, ‘suspected/alleged’, ‘rib/limb fractures’, ‘assaults’) to the original Confirmed CM list sensitivity is increased with a decrease in specificity (12 months around index date sensitivity, 61.1%[95% CI 56.9–65.2] specificity, 89.0%[95% CI 87.3–90.4]; present in records at any time sensitivity, 71.8%[95% CI 67.8–75.5] specificity, 85.0%[95% CI 83.2–86.7]).

### False positives

Using the Confirmed CM code list (prevalence subset) we incorrectly identified 203 out of 1652 individuals (12.2%) who had been clinically assessed as not being maltreated. Of these 199 had codes relating to child protection and 29 had confirmed maltreatment codes (either current or historical e.g., ‘Physical abuse’). The most used codes were ‘13IM. Child on protection register’ (n = 142) and ‘13IO. Child removed from protection register’ (n = 90).

### False negatives

A total of 254 of the 553 clinically confirmed cases were not identified using our algorithm. Of these 78 (30.7%) had at least one of the ‘Possible CM’ codes. The most frequently occurring of these codes was ‘U3… [x] assault’, followed by ‘13IB0 child in foster care’.

Of the remaining 175 false negatives the most used codes were administrative codes ‘e.g., notes summary on computer’ or routine codes such as inoculations, or paracetamol prescriptions. Other frequently used codes included ‘letter from specialist’, ‘seen in paediatric clinic’, and codes related to chest infections (e.g. ‘chest infection NOS’).

There were codes indicating hospital or emergency department attendance (e.g. ‘seen in hospital casualty’ or ‘emergency hospital admission’) without specific mention of maltreatment.

### Validation exercise 2: Hospital admissions data

Results of the validation exercise in hospital admissions data are shown in Table 3. This includes sensitivity analysis accounting for the impact of adding or removing various groups of codes. Sensitivity of Confirmed CM codes was lower in hospital admissions data than in primary care ranging from 9.4 (95% CI 7.2–12.2) to 27.8 (95% CI 24.2–31.8). Specificity was high ranging from 96.4 (95% CI 95.3–97.2) to 99.4 (95% CI 98.9 – 99.7).

**Table 3:** Sensitivity (95% CI), Specificity (95% CI), PPV(95% CI) NPV(95% CI) of code lists for child maltreatment included sensitivity analysis of additional and exclusion of categories of.

	Sensitivity		Specificity		PPV^a^		NPV^a^	
	Time 1^b^	Time 2^c^	Time 1^b^	Time 2^c^	Time 1^b^	Time 2^c^	Time 1^b^	Time 2^c^
Confirmed CM^d^	9.4 (7.2–12.2)	10.3 (8.0–13.2)	99.4 (98.9–99.7)	99.0 (98.3–99.4)	76.6 (74.8–78.4)	76.7 (74.9–78.5)	76.6 (74.8–78.4)	76.7 (74.9–78.5)
‘Other problems with primary support group' codes added	14.5 (11.7–17.7)	18.1 (15.0–21.6)	98.4 (97.6–98.9)	97.5 (96.6–98.2)	74.8 (65.3–82.4)	70.9 (62.6–78.1)	77.5 (75.6–79.2)	78.1 (76.2–79.8)
‘Suspected/alleged maltreatment' codes added	10.7 (8.3–13.6)	11.8 (9.2–14.8)	99.4 (98.9–99.7)	99.0 (98.3–99.4)	85.5 (74.5–92.5)	79.3 (68.6–87.1)	76.9 (75.0–78.6)	77.0 (75.2–78.8)
‘Rib/limb fractures/dislocations' codes added	11.9 (9.4–15.0)	14.5 (11.7–17.7)	99.0 (98.3–99.4)	98.1 (97.3–98.7)	79.5 (69.0–87.3)	72.1 (62.6–80.0)	77.0 (75.2–78.8)	77.4 (75.5–79.2)
‘Assaults' codes added	12.8 (10.2–16.0)	14.8 (12.0–18.1)	99.1 (98.5–99.5)	98.5 (97.8–99.0)	82.6 (72.5–89.6)	77.4 (68.0–84.7)	77.3 (75.4–79.0)	77.6 (75.7–79.3)
Inclusion of all additional codes	23.7 (20.2–27.5)	27.8 (24.2–31.8)	97.2 (96.2–97.9)	96.4 (95.3–97.2)	73.6 (66.4–79.8)	72.0 (65.4–77.8)	79.2 (77.3–80.9)	80.0 (78.1–81.7)

^a^PPV and NPV based on prevalence in validation population.

^b^Hospital admission within 12 months before or after index date.

^c^Hospital admission at any time.

^d^Confirmed CM codes encompass maltreatment related syndromes, genital mutilation and prostitution. Note that child protection codes are not available in hospital admissions data.

### False positives

Using the CM code list, we incorrectly identified 10 out of 1652 individuals (0.6%) who had been clinically assessed as not being maltreated. Of these almost all (numbers masked for confidentiality) had a code for; maltreatment syndromes’ (T74) alongside codes for ‘other maltreatment’ (Y07) and ‘neglect and abandonment’ (Y06).

### False negatives

A total of 501 of the 553 clinically confirmed cases were not identified using our algorithm. Of these 235 were admitted to hospital within the 12 months either side of the index date.

Of these 61 (26.0%) had at least one of the ‘Possible CM’ codes. The most commonly occurring of these were codes for Assaults (n = 61) followed by Z638 ‘Other specified problems related to primary support group (excl. maltreatment syndromes, negative life events in childhood and upbringing; n = 28), and Fractures/dislocations/rib fractures (n = 14).

Of the remaining 174 false negatives the most used codes were for ‘Injury, poisoning and other consequences of external causes’. The most used single codes were S00 ‘superficial injury of head’, X59 ‘Exposure to unspecified factor’ and K02 ‘dental caries’. It appears that coding in hospital admissions may be more focused on the injury in need of treatment than on recording the presence of maltreatment. Also of note are the absence of child protection and social care codes that account for a large proportion of correctly identified cases in primary care.

### Validation exercise 3: linking GP and admissions data

Combining GP and hospital admissions data to identify CM improved sensitivity slightly compared to using either dataset individually (12 months around index date, GP only 43.0 [95% CI 38.9–473], admissions only 9.4 [95% CI 7.2–12.2], GP and admissions 47.0 [95% CI 42.8 -51.3]; Record at any time GP only 54.1[95% 49.8–58.3], admissions only 10.3[95% CI 8.0–13.2], GP and admissions 57.7[95% CI 53.4–61.8] Table 4). There was only a slight decrease in specificity. Similar results were seen when looking at the Possible CM codes (Table 4).

**Table 4:** Sensitivity (95% CI), specificity (95% CI), PPV(95% CI) NPV(95% CI) of code lists for child maltreatment included sensitivity analysis of additional and exclusion of categories of indicative/at-risk codes in hospital admission data.

	Sensitivity		Specificity		PPV^a^		NPV^a^	
	Time 1^b^	Time 2^c^	Time 1^b^	Time 2^c^	Time 1^b^	Time 2^c^	Time 1^b^	Time 2^c^
Confirmed CM^d^	47.0 (42.8–51.3)	57.3 (53.1–61.5)	91.2 (89.7–92.5)	87.6 (85.9–89.1)	64.0 (59.1–68.7)	60.7 (56.4–64.9)	83.7 (81.9–85.4)	86.0 (84.2–87.6)
Confirmed CM and possible CM combined	67.6 (63.5–71.5)	75.9 (72.1–79.4)	88.0 (86.3–89.5)	84.2 (82.3–85.9)	65.3 (61.2–69.1)	61.7 (57.9–65.3)	89.0 (87.4–90.5)	91.3 (89.7–92.6)

^a^PPV and NPV based on prevalence in validation population.

^b^Hospital admission or GP record within 12 months before or after index date.

^c^Hospital admission or GP record at any time.

^d^Confirmed CM codes encompass maltreatment related syndromes, genital mutilation, and prostitutions. Note that child protection codes are not available in hospital admissions data.

**Figure 1:**
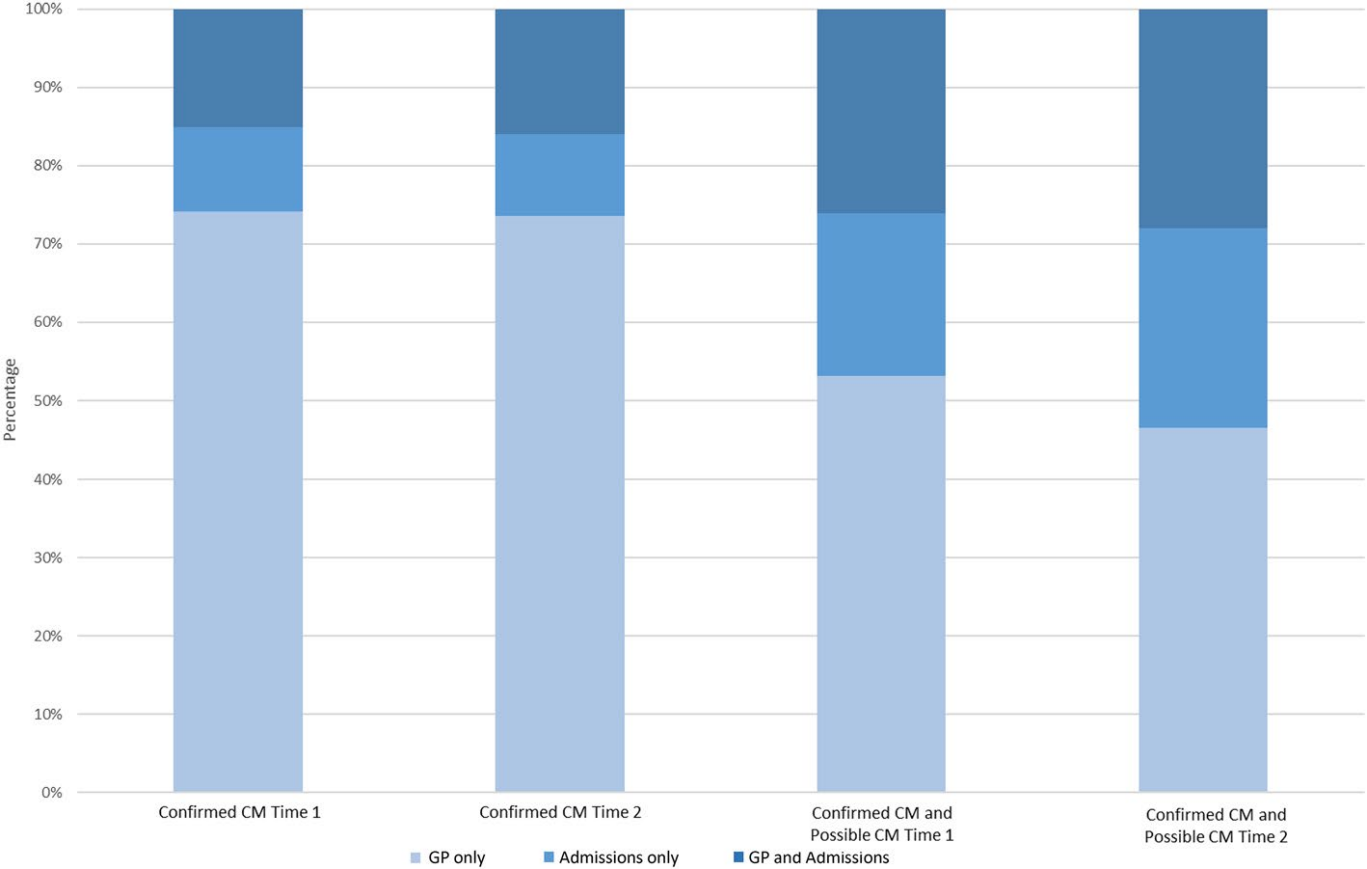
Percentage of cases picked up in each healthcare setting stratified by CM/risk possible and follow-up period^a^. (a) Time 1 refers to 12 months either side of index date; Time 2 at any point during follow-up period.

When looking at Confirmed CM 82.1% of cases were found identified in GP data only, 17.9% in admissions data only and 11.6% in both GP and admissions data (Fig. 1). When looking at the Possible CM codes the proportion identified in admissions data increased (GP only 62.4%; admissions only 34.4%; GP and admissions 37.6%).

### Incidence and prevalence over time

Incidence of Confirmed CM in both GP and admissions data was comparable between sexes. Incidence decreased with increasing age with the highest incidence in those aged < 1 year (IRR GP 3.5[95% CI 3.1–3.9]; admissions 5.6[95% CI 4.2–7.5] 15–17 years as a reference group). Incidence was highest in the most deprived quintiles with more than five times the risk in GP data and six times the risk in admissions (IRR GP 5.4[95% CI 5.0–5.9]; admissions 6.1[95% CI 4.4–8.4]). Individuals with no deprivation data were also at increased risk (Tables 5 and 6).

**Table 5:** GP Events, incidence^a^ per 1000 PYAR (95% CI), IRR^b^ (95% CI)^c^ of confirmed CM and possible CM by year, sex, age group and deprivation quintile.

Variable		Confirmed CM			Possible CM			Confirmed and possible CM		
		Events	Incidence (95% CI)	IRR (95% CI)	Events	Incidence (95% CI)	IRR (95% CI)	Events	Incidence (95% CI)	IRR (95% CI)
Year	2004	1429	3.1 (3.0–3.3)	Reference (p < 0.001)	2507	5.5 (5.3–5.7)	Reference (p < 0.001)	3570	7.8 (7.6–8.1)	Reference (p < 0.001)
	2005	1355	2.9 (2.8–3.1)	0.9 (0.8–1.1)	2809	6.1 (5.9–6.3)	1.1 (1.0–1.3)	3689	8.0 (7.7–8.2)	1.0 (0.9–1.1)
	2006	1639	3.5 (3.4–3.7)	1.1 (1.0–1.3)	3262	7.0 (6.8–7.3)	1.3 (1.1–1.4)	4279	9.2 (8.9–9.5)	1.2 (1.0–1.3)
	2007	1726	3.7 (3.5–3.9)	1.2 (1.0–1.3)	3502	7.6 (7.3–7.8)	1.4 (1.2–1.5)	4570	9.9 (9.6–10.1)	1.2 (1.1–1.4)
	2008	1815	3.9 (3.7–4.1)	1.2 (1.1–1.4)	3861	8.3 (8.0–8.5)	1.5 (1.3–1.6)	5004	10.7 (10.4–11.0)	1.3 (1.2–1.5)
	2009	2013	4.3 (4.1–4.5)	1.3 (1.2–1.5)	4317	9.2 (9.0–9.5)	1.6 (1.5–1.8)	5473	11.7 (11.4–12.0)	1.5 (1.3–1.6)
	2010	2071	4.5 (4.3–4.7)	1.4 (1.2–1.5)	4594	9.9 (9.6–10.2)	1.7 (1.5–1.9)	5834	12.6 (12.2–12.9)	1.5 (1.4–1.7)
	2011	2245	4.8 (4.6–5.0)	1.5 (1.3–1.7)	4706	10.1 (9.9–10.4)	1.8 (1.6–2.0)	6038	13.0 (12.7–13.3)	1.6 (1.4–1.8)
	2012	2481	5.3 (5.1–5.5)	1.6 (1.4–1.8)	5190	11.2 (10.9–11.5)	1.9 (1.7–2.2)	6604	14.2 (13.9–14.5)	1.7 (1.6–1.9)
	2013	2188	4.7 (4.5–4.9)	1.4 (1.3–1.6)	4944	10.6 (10.3–10.9)	1.9 (1.7–2.1)	6181	13.3 (13.0–13.6)	1.6 (1.5–1.8)
	2014	2493	5.3 (5.1–5.5)	1.6 (1.4–1.9)	5331	11.4 (11.1–11.7)	2.0 (1.8–2.2)	6699	14.3 (14.0–14.7)	1.8 (1.6–2.0)
	2015	2580	5.5 (5.3–5.7)	1.7 (1.5–1.9)	5326	11.3 (11.0–11.6)	2.0 (1.8–2.2)	6748	14.4 (14.0–14.7)	1.8 (1.6–2.0)
	2016	2484	5.3 (5.1–5.5)	1.6 (1.4–1.8)	5591	11.9 (11.5–12.2)	2.1 (1.8–2.3)	6976	14.8 (14.4–15.1)	1.8 (1.6–2.0)
	2017	2437	5.1 (4.9–5.3)	1.5 (1.3–1.8)	5514	11.6 (11.3–11.9)	2.0 (1.8–2.3)	6894	14.5 (14.2–14.9)	1.8 (1.6–2.0)
	2018	2180	4.6 (4.5–4.8)	1.4 (1.2–1.6)	5201	11.1 (10.8–11.4)	1.9 (1.7–2.2)	6468	13.8 (13.5–14.1)	1.7 (1.5–1.9)
	2019	2050	4.3 (4.2–4.5)	1.3 (1.1–1.5)	5153	10.9 (10.6–11.2)	1.9 (1.7–2.1)	6429	13.6 (13.3–13.9)	1.6 (1.5–1.9)
	2020^d^	1192	3.5 (3.3–3.7)	1.1 (1.0–1.3)	2272	6.6 (6.4–6.9)	1.2 (1.1–1.4)	3033	8.8 (8.5–9.2)	1.2 (1.0–1.3)
Gender	Male	17,257	4.3 (4.2–4.4)	Reference (p = 0.037)	38,580	9.6 (9.5–9.7)	Reference (p = 0.22)	48,775	12.2 (12.1–12.3)	Reference (p = 0.333)
	Female	17,121	4.5 (4.4–4.6)	1.0 (1.0–1.1)	35,500	9.3 (9.2–9.4)	1.0 (0.9–1.0)	45,714	12.0 (11.9–12.1)	1.0 (1.0–1.0)
Age group	15–17 years	3044	2.6 (2.5–2.7)	Reference (p < 0.001)	7810	29.2 (28.8–29.7)	Reference (p < 0.001)	10,006	8.6 (8.4–8.8)	Reference (p < 0.001)
	10–14 years	7157	3.2 (3.1–3.3)	1.2 (1.1–1.4)	15,704	11.1 (10.9–11.2)	1.0 (1.0–1.1)	19,868	8.9 (8.8–9.0)	1.0 (1.0–1.1)
	5–9 years	8389	3.9 (3.8–4.0)	1.4 (1.3–1.6)	14,688	6.8 (6.7–6.9)	1.0 (0.9–1.0)	19,589	9.1 (8.9–9.2)	1.0 (1.0–1.1)
	1–4 years	8842	5.4 (5.3–5.5)	1.9 (1.7–2.2)	18,189	7.0 (6.9–7.1)	1.6 (1.5–1.6)	23,214	14.1 (14.0–14.3)	1.6 (1.5–1.7)
	< 1 years	6946	11.5 (11.2–11.7)	3.5 (3.1–3.9)	17,689	6.7 (6.6–6.9)	3.5 (3.3–3.7)	21,812	36.0 (35.6–36.5)	3.3 (3.1–3.5)
Deprivation	Least deprived	1895	1.3 (1.3–1.4)	Reference (p < 0.001)	6745	4.7 (4.6–4.8)	Reference (p < 0.001)	8015	5.6 (5.5–5.7)	Reference (p < 0.001)
	2	2955	2.4 (2.3–2.5)	1.8 (1.6–2.0)	7873	6.5 (6.3–6.6)	1.4 (1.3–1.4)	9697	8.0 (7.8–8.1)	1.4 (1.3–1.5)
	3	4551	3.2 (3.1–3.3)	2.4 (2.2–2.6)	10,355	7.3 (7.2–7.5)	1.5 (1.5–1.6)	12,990	9.2 (9.0–9.4)	1.6 (1.5–1.7)
	4	7146	4.6 (4.5–4.7)	3.4 (3.1–3.7)	14,603	9.5 (9.3–9.6)	1.9 (1.9–2.0)	18,759	12.2 (12.0–12.4)	2.1 (2.0–2.2)
	Most deprived	13,489	7.5 (7.4–7.6)	5.4 (5.0–5.9)	23,751	13.2 (13.0–13.3)	2.7 (2.6–2.8)	31,468	17.5 (17.3–17.7)	3.0 (2.9–3.1)
	Unknown	4342	10.6 (10.3–10.9)	4.7 (4.2–5.2)	10,753	26.3 (25.8–26.8)	2.8 (2.6–3.0)	13,560	33.1 (32.6–33.7)	3.1 (2.9–3.3)

^a^Event refers to an individual presenting with Confirmed CM/Possible CM who has not had a record of this in the previous 12 months.

^b^Adjusted for calendar year, sex, age and deprivation.

^c^Based on Wald test.

^d^Data collection to 10.10.2022; denominators adjusted.

**Table 6:** Hospital admissions, incidence^a^ per 1000 PYAR (95% CI), IRR^b^ (95% CI)^c^ of confirmed CM and possible CM by year, sex, age group and deprivation quintile.

Variable		Confirmed CM			Possible CM			Confirmed and possible CM		
		Events	Incidence (95% CI)	IRR (95% CI)	Events	Incidence (95% CI)	IRR (95% CI)	Events	Incidence (95% CI)	IRR (95% CI)
Year	2004	31	0.1 (0.0–0.1)	Reference (p < 0.001)	1231	2.7 (2.6–2.9)	Reference (p < 0.001)	1250	2.7 (2.6–2.9)	Reference (p < 0.001)
	2005	39	0.1 (0.1–0.1)	1.2 (0.8–1.9)	1326	2.9 (2.7–3.0)	1.1 (0.9–1.2)	1352	2.9 (2.8–3.1)	1.1 (0.9–1.2)
	2006	60	0.1 (0.1–0.2)	1.9 (1.3–2.7)	1426	3.1 (2.9–3.2)	1.1 (1.0–1.3)	1460	3.1 (3.0–3.3)	1.1 (1.0–1.3)
	2007	57	0.1 (0.1–0.2)	1.8 (1.2–2.6)	1438	3.1 (2.9–3.3)	1.1 (1.0–1.3)	1474	3.2 (3.0–3.3)	1.2 (1.0–1.3)
	2008	71	0.2 (0.1–0.2)	2.1 (1.5–3.1)	1428	3.1 (2.9–3.2)	1.1 (1.0–1.3)	1465	3.1 (3.0–3.3)	1.1 (1.0–1.3)
	2009	79	0.2 (0.1–0.2)	2.4 (1.6–3.6)	1411	3.0 (2.9–3.2)	1.1 (1.0–1.3)	1465	3.1 (3.0–3.3)	1.2 (1.0–1.3)
	2010	79	0.2 (0.1–0.2)	2.4 (1.6–3.5)	1268	2.7 (2.6–2.9)	1.0 (0.9–1.2)	1321	2.8 (2.7–3.0)	1.1 (0.9–1.2)
	2011	57	0.1 (0.1–0.2)	1.7 (1.1–2.5)	1211	2.6 (2.5–2.8)	1.0 (0.9–1.1)	1248	2.7 (2.5–2.8)	1.0 (0.9–1.1)
	2012	38	0.1 (0.1–0.1)	1.1 (0.8–1.7)	1052	2.3 (2.1–2.4)	0.8 (0.8–1.0)	1076	2.3 (2.2–2.5)	0.9 (0.8–1.0)
	2013	39	0.1 (0.1–0.1)	1.2 (0.7–1.8)	1125	2.4 (2.3–2.6)	0.9 (0.8–1.0)	1149	2.5 (2.3–2.6)	0.9 (0.8–1.0)
	2014	31	0.1 (0.0–0.1)	0.9 (0.6–1.5)	1192	2.6 (2.4–2.7)	1.0 (0.9–1.1)	1214	2.6 (2.5–2.7)	1.0 (0.9–1.1)
	2015	24	0.1 (0.0–0.1)	0.7 (0.4–1.2)	1115	2.4 (2.2–2.5)	0.9 (0.8–1.0)	1134	2.4 (2.3–2.6)	0.9 (0.8–1.0)
	2016	47	0.1 (0.1–0.1)	1.4 (0.9–2.1)	1135	2.4 (2.3–2.5)	0.9 (0.8–1.0)	1164	2.5 (2.3–2.6)	0.9 (0.8–1.0)
	2017	32	0.1 (0.0–0.1)	0.9 (0.6–1.5)	1159	2.4 (2.3–2.6)	0.9 (0.8–1.0)	1182	2.5 (2.3–2.6)	0.9 (0.8–1.1)
	2018	36	0.1 (0.1–0.1)	1.1 (0.7–1.7)	987	2.1 (2.0–2.2)	0.8 (0.7–0.9)	1017	2.2 (2.0–2.3)	0.8 (0.7–0.9)
	2019	46	0.1 (0.1–0.1)	1.3 (0.9–2.0)	1085	2.3 (2.2–2.4)	0.9 (0.8–1.0)	1121	2.4 (2.2–2.5)	0.9 (0.8–1.0)
	2020^d^	20	0.1 (0.0–0.1)	0.9 (0.5–1.5)	478	1.4 (1.3–1.5)	0.5 (0.5–0.6)	490	1.4 (1.3–1.6)	0.5 (0.5–0.6)
Gender	Male	412	0.1 (0.1–0.1)	Reference (p = 0.549)	13,034	3.3 (3.2–3.3)	Reference (p < 0.001)	13,292	3.3 (3.3–3.4)	Reference (p < 0.001)
	Female	374	0.1 (0.1–0.1)	1.0 (0.8–1.1)	7033	1.9 (1.8–1.9)	0.6 (0.5–0.6)	7290	1.9 (1.9–2.0)	0.6 (0.6–0.6)
Age group	15–17 years	65	0.1 (0.0–0.1)	Reference (p < 0.001)	4903	4.2 (4.1–4.3)	Reference (p < 0.001)	4947	4.2 (4.1–4.4)	Reference (p < 0.001)
	10–14 years	180	0.1 (0.1–0.1)	1.4 (1.1–1.9)	6198	2.8 (2.7–2.8)	0.7 (0.6–0.7)	6320	2.8 (2.8–2.9)	0.7 (0.6–0.7)
	5–9 years	132	0.1 (0.1–0.1)	1.1 (0.8–1.5)	4079	1.9 (1.8–1.9)	0.5 (0.4–0.5)	4170	1.9 (1.9–2.0)	0.5 (0.4–0.5)
	1–4 years	196	0.1 (0.1–0.1)	2.1 (1.5–2.8)	3129	1.9 (1.8–2.0)	0.5 (0.4–0.5)	3264	2.0 (1.9–2.1)	0.5 (0.4–0.5)
	< 1 years	213	0.4 (0.3–0.4)	5.6 (4.2–7.5)	1758	2.9 (2.8–3.0)	0.6 (0.5–0.6)	1881	3.1 (3.0–3.3)	0.6 (0.6–0.7)
Deprivation	Least deprived	43	0.0 (0.0–0.0)	Reference (p < 0.001)	2989	2.1 (2.0–2.2)	Reference (p < 0.001)	3020	2.1 (2.0–2.2)	Reference (p < 0.001)
	2	67	0.1 (0.0–0.1)	1.8 (1.2–2.7)	2827	2.3 (2.2–2.4)	1.1 (1.0–1.2)	2873	2.4 (2.3–2.5)	1.1 (1.0–1.2)
	3	80	0.1 (0.0–0.1)	1.9 (1.3–2.7)	3372	2.4 (2.3–2.5)	1.2 (1.1–1.2)	3416	2.4 (2.3–2.5)	1.2 (1.1–1.2)
	4	152	0.1 (0.1–0.1)	3.2 (2.2–4.6)	4102	2.7 (2.6–2.7)	1.3 (1.2–1.4)	4206	2.7 (2.6–2.8)	1.3 (1.2–1.4)
	Most deprived	340	0.2 (0.2–0.2)	6.1 (4.4–8.4)	5315	2.9 (2.9–3.0)	1.4 (1.4–1.6)	5544	3.1 (3.0–3.2)	1.5 (1.4–1.6)
	Unknown	104	0.3 (0.2–0.3)	3.6 (2.5–5.3)	1462	3.6 (3.4–3.8)	1.8 (1.6–2.0)	1523	3.7 (3.5–3.9)	1.8 (1.6–2.0)

^a^Event refers to an individual presenting with CM/Possible CM who has not had a record of this in the previous 12 months.

^b^Adjusted for calendar year, sex, age and deprivation.

^c^Based on Wald test.

^d^Data collection to 10.10.2022; denominators adjusted.

When exploring GP contacts for Possible CM demographic indices broadly mirrored that seen for Confirmed CM with little difference between sexes and a decreasing incidence with increasing age (IRR < 1 year 3.5 [95% CI 3.3–3.7] 15–17 years as reference group). An increase in incidence was seen with increasing deprivation, however this was smaller for Possible CM than Confirmed CM with over double the rate in the most deprived compared with the least deprived quintiles (IRR 2.6 [95% CI 2.6–2.8]). Those with unknown deprivation were also at increased risk.

Admissions related to Possible CM demonstrated differing demographic indices than the Confirmed CM with around double the admissions in males than in females and an increase in incidence rate with increasing age (Table 6). Further exploratory analysis revealed that this was attributable to assaults and fractures, rates of which increase with increasing age.

**Figure 2:**
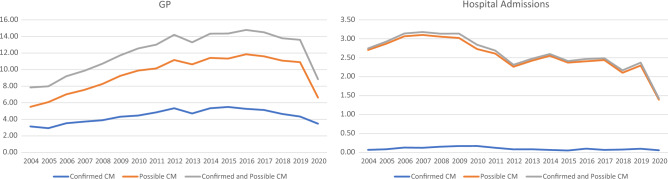
Incidence per 1000 PYAR of confirmed CM and possible CM by setting over time.

Incidence of GP events for Confirmed CM increased from 3.1 (95% CI 3.0–3.3) cases per 1000 PYAR in 2004 to 4.3(95% CI 4.2–4.5) in 2019 (IRR 1.3 [95% CI 1.1–1.5]) with a decrease seen in 2020 (Fig. 2; Table 5). Trends over time were similar for Possible CM although the overall rate over time was higher (IRR 2019 1.9 [95% CI 1.7–2.1]).

Incidence of Confirmed CM related admissions remained low throughout the study period with numbers with less than 80 admissions per year. Admissions for Possible CM initially increased from 2.7 (95% CI 2.6–2.9) in 2004 to 3.1 (95% CI 2.9–3.2) cases per 1000 PYAR I n 2008 (IRR 2008 1.1 [95% CI 0.0–1.3]). Cases then decreased from 2008 onwards with significant decrease in 2020 (IRR 2019 0.9 [95% CI 0.8–1.0]).

Similar trends for both GP contacts and admissions were seen for prevalence (Supplementary File 6 Tables S1 and S2).

## Discussion

### Main findings

This study demonstrates the creation and first external validation of codes and algorithms to identify cases of CM from routinely collected healthcare data. Sensitivity was higher than that identified by previously published CM code lists. We utilised the validated code lists and found an increase in the incidence and prevalence of both Confirmed CM and Possible CM over time in GP data with a low rate over time of hospital admissions related to Confirmed CM.

We linked a clinically assessed hospital-based CM cohort to cases identified in GP and hospital admissions records to assess the sensitivity, specificity and PPV NPV. Using Confirmed CM codes the algorithm performed with high specificity minimising the proportion of incorrectly identified cases, an important factor for most cohort and case control studies.. The difference between datasets in the ability to identify maltreatment was highlighted, with the majority of cases detected in primary care rather than hospital admissions. Of note, the proportion of cases identified exclusively in admissions data was higher for Possible CM codes than confirmed CM. The true extent of CM is difficult to establish in routine data due to the complexity of the attendance, recognition, recording and coding of maltreatment.

Sensitivity analyses were conducted to encompass a broader range of codes that may indicate maltreatment or individuals who are vulnerable or at risk. This improves sensitivity with a small negative impact on specificity. However, the nature of these codes means that their use should be considered on a study-by-study basis. False negatives frequently had codes for ‘other social services’ (e.g., child in care) and codes for assaults. While these codes may indicate risk and potential maltreatment, this may not always be the case. For example, in the case of social services codes, children may be involved with social services for a number of reasons including child or parental illness/disability unrelated to maltreatment. These individuals may have many co-occurring risk factors and appear similar to those who have codes for maltreatment in large databases of routinely collected data. The care, support and resources needed will be unique to circumstances and grouping these individuals together for research may not be appropriate. Similarly, codes for assault may not always indicate maltreatment. Consideration could be given to apply age constraints to these codes dependent on the study (e.g., assault codes only for those aged under 5 or under 10 years). The ‘Possible CM’ codes are comprehensive, but cannot be reliably used for case ascertainment, without further evidence to substantiate. Further work, through data learning techniques may prove fruitful to improve performance by combining codes, for instance code terms such as ‘maternal concern’ along with ‘emergency admissions to hospital’.

While some iterations of previously published code lists performed with high sensitivity, care must be taken for inclusion of codes that do not indicate maltreatment (e.g., ‘parental benefits’ or ‘self-neglect’). Many of these codes may indicate a potentially vulnerable child with similar risk factors and co-morbidities, however these represent distinct groups of individuals who require different types and levels of support. The importance of care in selecting and validating codes for maltreatment is emphasized.

There was an increase in both incidence and prevalence of Confirmed CM and Possible CM codes from 2004 to 2019 in primary care, with the largest increase seen in Possible CM codes which more than doubled over time. This could reflect a genuine increase, or an increase in GP coding and recognition of vulnerable children. Rates of both CM and Possible CM codes were comparable between sexes, decreased with increasing age and were highest in the most deprived areas. This was most notable for Confirmed CM codes with more than five times the incidence in the most deprived compared with the least deprived areas.

Rates of hospital admissions for Confirmed CM remained at a low rate over time, with an initial increase in Possible CM admissions and a decrease from 2009 onwards.

### False positives

Most false positives in GP records were identified from child protection codes. However, without these codes sensitivity is poor, picking up only around one fifth of cases. Child protection is the social services response to harm to a child and it seems reasonable to include these in any list of codes examining maltreatment. These CYP had the same confirmed CM codes recorded in their GP records as the confirmed cases, given this, it would be difficult to further improve specificity. These children may be at high risk of maltreatment, as possible CM is suggested within their medical records, however insufficient evidence of CM was found on the day of assessment.

### False negatives

Around half of the clinically confirmed cases were missed using the Confirmed CM code list in GP records. All children within the CVCM cohort would have been assessed because they were considered ‘at risk’ of or a ‘victim of’ CM, therefore all these children would be more likely to have maltreatment-related codes recorded in their medical records than would perhaps be the case for a random sample. We found (30.7%) of these cases did have at least one Possible CM code recorded in their primary care records. Future application of machine learning techniques using the Possible CM code lists may identify combinations of codes that improves sensitivity, with minimal detriment to specificity, to tease apart ‘confirmed’ from ‘possible’ cases and to optimise performance.

Around 90% of cases were missed using admissions data. Of those missed around half were not admitted to hospital in the 12 months either side of their maltreatment assessment date and as such cannot be captured by this data. The narrower coding framework used in hospital admissions also limits recording of maltreatment with child protection and social care codes absent. Coding in hospital admissions is focused on the injury being treated and not on whether this was the result of maltreatment. While specificity was high the under-reporting of CM in admissions data must be acknowledged in any future studies.

### Comparison to the literature

Incidence rates fall between those reported in two similar studies using routinely collected primary care data conducted in the UK^18,19^. These differences are likely attributable to the choice of codes employed to identify cases of maltreatment, which illustrates the need for standardisation in definitions and subsequent validation. Rates were higher than those reported by Chandan et al.^18^ most likely due to the addition of an extensive list of child protection codes in our algorithm. Rates using the confirmed CM list in the current study are lower than those reported by Woodman et al.^19^, however they included a wider set of codes, such as ‘out of home care’ and ‘social care’ codes, which we excluded from our Confirmed CM code list but, were present in our Possible CM code lists (incidence using this list was comparable to that found by Woodman et al.^19^). The findings from all three studies are in agreement that the incidence rates recorded in primary care underestimate the true rates present within the community, although incidence rates for CM reported in the current study are in keeping with ONS data on number of children in Wales subject to a child protection plan^7^.

The increase in CM over time as recorded in primary care is supported by previous research with routinely collected GP data^18,19^. The factors driving these increases in recordings in primary care is less clear. It may be due to raised awareness and real improvements in recognition, responding and changing coding and reporting behaviour to record all concerns of CM^19^, as a result of policies and practice guidance notes published by UK National Institute for Health and Care Excellence (NICE: 2009. 2016, 2017^9^) General Medical Council (GMC, 2012, updated 2018) guidance notes^10^, National Society for the Prevention of Cruelty to Children (NSPCC collaboration, 2014^8^) and Public Health Wales (PHW, 2015^4^).

There have also been increases in child protection activity in recent years, but it is unclear whether this is because child protection services have become better at recognising and responding to maltreatment. An observational time-series study using official government agency and NSPCC data in England and Wales, found that the incidence of crimes against children, child protection registrations and children entering care had increased steeply between 2000 and 2016^31^. It is difficult to know whether this is part of a trend of increasing reporting, as opposed to rising levels of maltreatment within society. Further time series studies using national survey data may be needed to establish whether CM is becoming more common.

We found a decrease in recorded CM in primary care in 2020. This is in keeping with concerns that cases of abuse may have been missed due to restricted access to protective services during lockdowns and disruptions to usual safeguarding pathways^32,33^. There are reports of reduced contact with public sector organisations such as schools, hospitals and emergency services in the UK^34^ and reductions in children added to the child protection register in 2020^35^. Data from one county borough demonstrated the largest decrease in referrals in the youngest children (aged < 3 years)^35^. This is alongside reports of increased contact with child abuse helplines^36^. Therefore, there may have been a disparity between incidence in public services and incidence in the community.

Rates of CM in primary care were highest in those aged less than a year old, with older adolescents having the lowest rates. Increased GP awareness of maltreatment in younger children, particularly from health workers surveillance and lower consultation rates for older children may be responsible for these differences^19^. Younger children are more likely to come to the attention of children’s services, particularly the under 1's^7^. Fewer adolescents are placed on child protection registers than any other age group. This age group may be more at risk of maltreatment through lack of identification and protection measures^31^. Admissions for Possible CM were highest in 15–17 year olds, largely attributable to the higher rates of assaults and fractures/dislocations in older age groups. Further research is needed to explore the nature of admissions for assaults and fractures/dislocations in older age groups and whether these presentations may represent an opportunity to identify and support adolescents at risk of maltreatment.

Incidence of Confirmed CM was more than five times as high in the most deprived compared with the least deprived communities in primary care and more than six times as high in hospital admissions. Individuals with no deprivation data were also at increased risk compared with the least deprived quintiles. This finding has been reported in other studies^18,19^. The relationship between family poverty and the likelihood of a child experiencing maltreatment is already well established^13,37,38^.

### Strengths and limitations

This study utilised a large population level database and the creation of comprehensive code lists for CM. Extensive manual searching was conducted alongside analysis of missed cases. It appears that the codes identified are exhaustive and that sensitivity could not be improved by adding additional codes. This underscores the importance of understanding that healthcare records underestimate the true incidence of maltreatment in the community.

This study further highlights the strengths and limitations of the individual healthcare datasets, their utility in detecting CM, and the use of these datasets in combination for study of CM. The difference in coding systems mean that comparison of rates between healthcare settings may not be appropriate. Further research is needed to assess comparability with settings outside of the UK.

Future research also may look to explore combinations of codes or machine learning to explore patterns of healthcare utilization to better identify CM in healthcare data.

Routinely collected data have limitations for research purposes, and the quality and completeness of data vary across datasets. We have attempted to minimise the impact of this by only including GPs that meet standards for data quality and validating study code lists. CM not resulting in presentation to services or where CM is discussed but not recorded will not be captured here. This is a common feature of all studies using routine data. These data are a reflection of contacts with the healthcare system, not rates of CM in the community.

There is selection bias in the externally validated CVCM dataset, as it represents a cohort of suspected victims/at risk of CM. All these CYP are therefore more likely to have a code suggestive of maltreatment recorded within the medical records. This makes improving performance of the algorithm more challenging, as we are effectively attempting to distinguish between ‘confirmed’ and ‘possible’ maltreatment cases.

### Implications

The validation of codes and development of algorithms from routinely collected datasets that identify cases with high specificity are an important step in epidemiological research. These validated code lists will be applicable to other datasets of routinely collected data and the choice of algorithm will vary with study design. This standardisation is important for research purposes to better understand the true effect and consequences of CM. Around half of the cases missed in admissions data were not admitted to hospital and as such could not be picked up in admissions data. Records of maltreatment in GP data appeared much more frequently. This is likely a combination of the higher number of contacts with primary care, communication between GPs and hospital settings being recorded and the extensive coding nomenclature in GP settings, in particular the presence of child protection and social care codes. This makes GPs better placed to record CM. It is important to include this setting to identify cases where possible. Where CM is being explored in admissions data the limitations must be recognized with around 90% of cases likely to be missed, although specificity in this setting is high.

We add to a body of evidence that CM recorded in primary care data has been increasing and further demonstrate a decreased in recorded CM in 2020.The long-term consequences of this drop in recording of maltreatment during the pandemic and potential disparity with community rates are as yet unknown. This has significance for informing future policy surrounding protective public services.. Future research should seek to explore this, and additional support considered for vulnerable children who may not have been identified during the pandemic.

Individuals in more deprived areas were at markedly increased risk of maltreatment. Also of note is the increased risk of those where deprivation data is unknown. This may indicate unstable living arrangements. Higher rates of maltreatment in these individuals may indicate the need for additional support or service provision. Further research is required to explore how best to support the most deprived communities or individuals where living arrangements may be unstable.

## Conclusions

Through the validation and assessment of CM-related codes in healthcare records, we create a platform for future epidemiological research. Time-series analysis on CM population-based epidemiological surveys may be needed to establish whether increasing recognition of cases represents rising trends within the community or whether it is simply due to improvements in recognition and responses or a combination of both. Additional support should be considered for individuals from deprived communities and those who may not have been identified as vulnerable during the pandemic.

## Supplementary Information

Supplementary Figure S1.

Supplementary Information 2.

Supplementary Information 3.

Supplementary Information 4.

Supplementary Information 5.

Supplementary Tables.

## Data Availability

The data used in this study are available in the SAIL Databank at Swansea University (Swansea, UK) via the Adolescent Mental Health Data Platform, but as restrictions apply they are not publicly available. All proposals to use SAIL data are subject to review by an independent Information Governance Review Panel (IGRP). Before any data can be accessed, approval must be given by the IGRP. The IGRP gives careful consideration to each project to ensure proper and appropriate use of SAIL data. When access has been granted, it is gained through a privacy protecting safe haven and remote access system referred to as the SAIL Gateway. SAIL has established an application process to be followed by anyone who would like to access data via SAIL at https://saildatabank.com/data/apply-to-work-with-the-data/.

## Abbreviations

ACEs: Adverse childhood experiences
ADP: The adolescent mental health data platform
BMA: British Medical Association
CI: Confidence interval
CM: Childhood maltreatment
CSEW: Crime Survey for England and Wales
CVCM: Cardiff and Vale University Health Board Minimum Dataset for CM
CYP: Child and young people
GMC: The General Medical Council
GP: General practitioner
GPD: General practice dataset
IGRP: Information governance review panel
IR: Incidence rate
IRR: Incidence rate ratio
ISPCAN: The International Society for the Prevention of Child Abuse & Neglect
NICE: The National Institute for Health and Care Excellence
NRES: National Research Ethics Service
NSPCC: National Society for the Prevention of Cruelty to Children
ONS: Office of National Statistics
PEDW: Patient Episode Database Wales
PHW: Public Health Wales
PPV: Positive predictive value
PYAR: Person years at risk
SAIL: Secure anonymised information linkage
SQL: Structured query language
THIN: The health improvement network
UKSeRP: The UK Secure e-Research Platform
WDS: Welsh Demographic Service
WIMD: Welsh Index of Multiple Deprivation
WHO: The World Health Organisation

## References

[1] Gilbert R, Widom CS, Browne K, Fergusson D, Webb E, Janson S (2009). Burden and consequences of child maltreatment in high-income countries. Lancet.

[2] Preventing child maltreatment: a guide to taking action and generating evidence.: World Health Organisation and International Society for Prevention of Child Abuse and Neglect (2006) https://apps.who.int/iris/bitstream/handle/10665/43499/9241594365_eng.pdf?sequence=1 (Accessed 20 Dec 2022).

[3] Radford L, Corral S, Bradley C, Fisher HL (2013). The prevalence and impact of child maltreatment and other types of victimization in the UK: Findings from a population survey of caregivers, children and young people and young adults. Child Abuse Negl..

[4] Adverse Childhood Experiences and their impact on health-harming behaviours in the Welsh adult population: Public Health Wales NHS Trust (2015). http://www2.nphs.wales.nhs.uk:8080/PRIDDocs.nsf/7c21215d6d0c613e80256f490030c05a/d488a3852491bc1d80257f370038919e/$FILE/ACE%20Report%20FINAL%20(E).pdf. (Accessed 20 Dec 2022).

[5] Child abuse in England and Wales: January 2020: Office for National Statistics (2020) https://www.ons.gov.uk/peoplepopulationandcommunity/crimeandjustice/bulletins/childabuseinenglandandwales/january2020 (Accessed 20 Dec 2022).

[6] Everson MD, Smith JB, Hussey JM, English D, Litrownik AJ, Dubowitz H (2008). Concordance between adolescent reports of childhood abuse and Child Protective Service determinations in an at-risk sample of young adolescents. Child Maltreat..

[7] Child abuse extent and nature, England and Wales, year ending March 2019: Office for National Statistics (ONS) (2020) https://www.ons.gov.uk/peoplepopulationandcommunity/crimeandjustice/articles/childabuseextentandnatureenglandandwales/yearendingmarch2019 (Accessed 20 Dec 2022)

[8] The GP's role in responding to child maltretment: time for a rethink? An overview of policy, practice and research: NSPCC, RCGP, UCL Institute of Child Health and the University of Surrey (2014) https://www.ehcap.co.uk/content/sites/ehcap/uploads/NewsDocuments/219/RCGP-GP-Role-responding-to-child-maltreatment-July-2014-ashx.PDF (Accessed 20 Dec 2022)

[9] Child abuse and neglect: The National Institute for Health and Care Excellence (NICE) (2017) https://www.nice.org.uk/guidance/ng76/chapter/Recommendations#recognising-child-abuse-and-neglect (Accessed 20 Dec 2022).

[10] Protecting children and young people: The responsibilities of all doctors: General Medical Council; Updated (2018) https://www.gmc-uk.org/ethical-guidance/ethical-guidance-for-doctors/protecting-children-and-young-people (Accessed 20 Dec 2022).

[11] Gonzalez-Izquierdo A, Ward A, Smith P, Walford C, Begent J, Ioannou Y (2015). Notifications for child safeguarding from an acute hospital in response to presentations to healthcare by parents. Child Care Health Dev..

[12] Guthridge SL, Ryan P, Condon JR, Moss JR, Lynch J (2014). Trends in hospital admissions for conditions associated with child maltreatment, Northern Territory, 1999–2010. Med. J. Aust..

[13] Imran S, Cross C, Das SU (2019). Association between socioeconomic status and risk of hospitalization due to child maltreatment in the USA. J. Investig. Med..

[14] Lo CK, Ho FK, Chan KL, Wong WH, Wong RS, Chow CB (2018). Linking healthcare and social service databases to study the epidemiology of child maltreatment and associated health problems: Hong Kong's experience. J. Pediatr..

[15] McKenzie K, Scott DA (2011). Using routinely collected hospital data for child maltreatment surveillance: Issues, methods and patterns. BMC Public Health.

[16] Chandan JS, Thomas T, Gokhale KM, Bandyopadhyay S, Taylor J, Nirantharakumar K (2019). The burden of mental ill health associated with childhood maltreatment in the UK, using The Health Improvement Network database: A population-based retrospective cohort study. Lancet Psychiatry.

[17] Woodman J, Allister J, Rafi I, de Lusignan S, Belsey J, Petersen I (2012). A simple approach to improve recording of concerns about childmaltreatment in primary care records: Developing a quality improvement intervention. Br. J. Gen. Pract..

[18] Chandan JS, Gokhale KM, Bradbury-Jones C, Nirantharakumar K, Bandyopadhyay S, Taylor J (2020). Exploration of trends in the incidence and prevalence of childhood maltreatment and domestic abuse recording in UK primary care: A retrospective cohort study using 'the health improvement network' database. BMJ Open.

[19] Woodman J, Freemantle N, Allister J, de Lusignan S, Gilbert R, Petersen I (2012). Variation in recorded child maltreatment concerns in UK primary care records: A cohort study using The Health Improvement Network (THIN) database. PLoS ONE.

[20] Syed S, Gonzalez-Izquierdo A, Allister J, Feder G, Li L, Gilbert R (2022). Identifying adverse childhood experiences with electronic health records of linked mothers and children in England: A multistage development and validation study. Lancet Digit. Health..

[21] John A, McGregor J, Fone D, Dunstan F, Cornish R, Lyons RA (2016). Case-finding for common mental disorders of anxiety and depression in primary care: An external validation of routinely collected data. BMC Med. Inform. Decis. Mak..

[22] Ford DV, Jones KH, Verplancke JP, Lyons RA, John G, Brown G (2009). The SAIL Databank: Building a national architecture for e-health research and evaluation. BMC Health Serv. Res..

[23] Lyons RA, Jones KH, John G, Brooks CJ, Verplancke JP, Ford DV (2009). The SAIL databank: Linking multiple health and social care datasets. BMC Med. Inform. Decis. Mak..

[24] John A, Marchant A, McGregor J, Tan J, Hutchings H, Kovess V (2015). Recent trends in the incidence of anxiety and prescription of anxiolytics and hypnotics in children and young people: An e-cohort study. J. Affect. Disord..

[25] McKenzie K, Scott DA, Waller GS, Campbell M (2011). Reliability of routinely collected hospital data for child maltreatment surveillance. BMC Public Health.

[26] Schnitzer PG, Slusher PL, Kruse RL, Tarleton MM (2011). Identification of ICD codes suggestive of child maltreatment. Child Abuse Negl..

[27] Chandan JS, Gokhale KM, Bradbury-Jones C, Nirantharakumar K, Bandyopadhyay S, Taylor J (2020). Exploration of trends in the incidence and prevalence of childhood maltreatment and domestic abuse recording in UK primary care: A retrospective cohort study using ‘the health improvement network’ database. BMJ Open.

[28] Chandan JS, Okoth K, Gokhale KM, Bandyopadhyay S, Taylor J, Nirantharakumar K (2020). Increased cardiometabolic and mortality risk following childhood maltreatment in the United Kingdom. J. Am. Heart Assoc..

[29] Garza HH, Piper KE, Barczyk AN, Pérez A, Lawson KA (2021). Accuracy of ICD-10-CM coding for physical child abuse in a paediatric level I trauma centre. Inj. Prev..

[30] Rothman, K. J. Epidemiologic analysis with a programmable calculator. NIH Pub No 79-1649, 31 (National Institutes of Health, 1979).

[31] DegliEsposti M, Humphreys DK, Jenkins BM, Gasparrini A, Pooley S, Eisner M (2019). Long-term trends in child maltreatment in England and Wales, 1858–2016: An observational, time-series analysis. Lancet Public Health..

[32] Caron F, Plancq MC, Tourneaux P, Gouron R, Klein C (2020). Was child abuse underdetected during the COVID-19 lockdown?. Arch. Pediatr..

[33] Bhopal S, Buckland A, McCrone R, Villis AI, Owens S (2021). Who has been missed? Dramatic decrease in numbers of children seen for child protection assessments during the pandemic. Arch. Dis. Child..

[34] Lynn RM, Avis JL, Lenton S, Amin-Chowdhury Z, Ladhani SN (2021). Delayed access to care and late presentations in children during the COVID-19 pandemic: A snapshot survey of 4075 paediatricians in the UK and Ireland. Arch. Dis. Child..

[35] Rengasamy ER, Long SA, Rees SC, Davies S, Hildebrandt T, Payne E (2022). Impact of COVID-19 lockdown: Domestic and child abuse in Bridgend. Child Abuse Neglect..

[36] NSPCC The impact of the coronavirus pandemic on child welfare: Domestic abuse. 1–13 (2020) https://learning.nspcc.org.uk/research-resources/2020/coronavirus-insight-briefing-sexual-abuse.

[37] Sidebotham P, Heron J, Golding J, Team As (2002). Child maltreatment in the "Children of the Nineties:" Deprivation, class, and social networks in a UK sample. Child Abuse Negl..

[38] The relationship between poverty, child abuse and neglect: an evidence review: Joseph Rowntree Foundation (2016) https://www.jrf.org.uk/report/relationship-between-poverty-child-abuse-and-neglect-evidence-review (Accessed 20 Dec 2022).

