# Supplementary material for: An external validation of coding for childhood maltreatment in routinely collected primary and secondary care data
Source: Sci Rep. 2023 May 19;13:8138. doi: 10.1038/s41598-023-34011-3 (PMC10199091; doi:10.1038/s41598-023-34011-3)
Supplement: Supplementary file 1 — Supplementary Figure S1. [file 41598_2023_34011_MOESM1_ESM.pptx]

## Slide 1
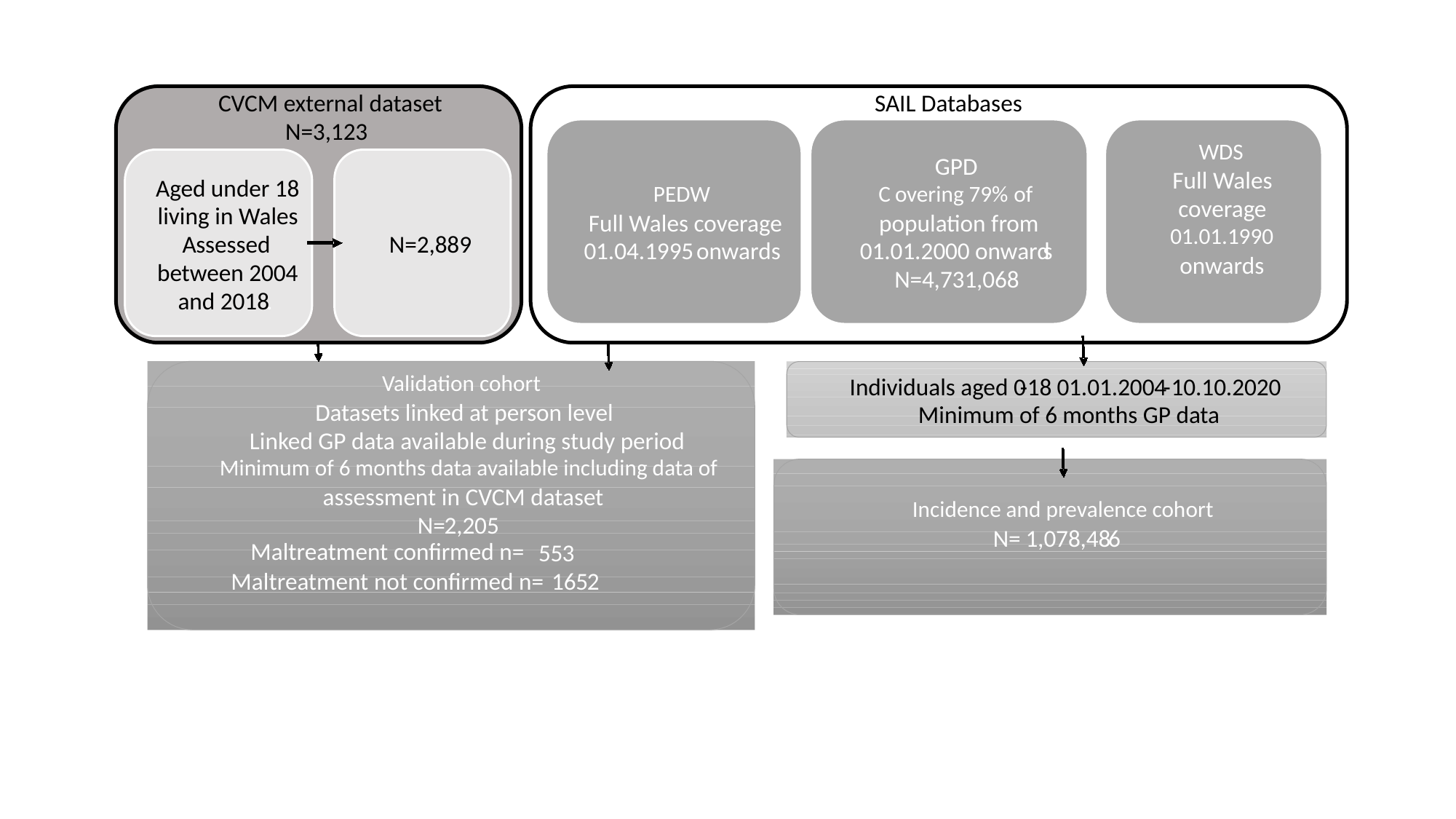

CVCM external dataset
SAIL Databases
N=3,123
WDS
GPD
Full Wales
Aged under 18
PEDW
C
overing 79%
of
coverage
living in Wales
Full Wales coverage
population from
01.01.1990
A
ssessed
N=2,889
01.04.1995
onwards
01.01.2000 onward
s
onwards
between 2004
N
=4,731,068
and 2018
.
Validation cohort
Individuals aged 0
-
18
01.01.2004
-
10.10.2020
Datasets linked at person level
Minimum of 6 months GP data
Linked GP data available during study period
Minimum of 6 months data available including data of
assessment in CVCM dataset
Incidence and prevalence cohort
N=
2,205
N=
1,078,48
6
Maltreatment confirmed n=
55
3
Maltreatment not confirmed n=
165
2
Figure S1 Creation of study cohorts
