# Supplementary material for: An external validation of coding for childhood maltreatment in routinely collected primary and secondary care data
Source: Sci Rep. 2023 May 19;13:8138. doi: 10.1038/s41598-023-34011-3 (PMC10199091; doi:10.1038/s41598-023-34011-3)
Supplement: Supplementary file 6 — Supplementary Tables. [file 41598_2023_34011_MOESM6_ESM.docx]

**Supplementary File 6 Supplementary Tables 1 and 2**

Supplementary Table 1 GP Events, prevalence^a^ per 1000 PYAR (95% CI), IRR^b^(95% CI)^c^ of confirmed CM and possible CM by year, sex, age group and deprivation quintile

|  | | | Confirmed CM | | | | | Possible CM | | | Confirmed and Possible CM | | |
| --- | --- | --- | --- | --- | --- | --- | --- | --- | --- | --- | --- | --- | --- |
| Variable |  | Events | | Incidence  (95% CI) | IRR (95% CI) | Events | Incidence  (95% CI) | | IRR  (95% CI) | Events | | Incidence  (95% CI) | IRR (95% CI) |
| Year | 2004 | 1808 | | 4.0(3.8-4.2) | Reference (p<.001) | 2665 | 5.9(5.6-6.1) | | Reference (p<.001) | 4071 | | 8.9(8.7-9.2) | Reference (p<.001) |
|  | 2005 | 1951 | | 4.2(4.0-4.4) | 1.1(0.9-1.2) | 3033 | 6.6(6.3-6.8) | | 1.1(1.0-1.3) | 4425 | | 9.6(9.3-9.9) | 1.1(1.0-1.2) |
|  | 2006 | 2185 | | 4.7(4.5-4.9) | 1.2(1.0-1.3) | 3541 | 7.6(7.4-7.9) | | 1.3(1.1-1.5) | 5066 | | 10.9(10.6-11.2) | 1.2(1.1-1.3) |
|  | 2007 | 2376 | | 5.1(4.9-5.3) | 1.3(1.1-1.4) | 3819 | 8.2(8.0-8.5) | | 1.4(1.2-1.5) | 5471 | | 11.8(11.5-12.1) | 1.3(1.2-1.4) |
|  | 2008 | 2619 | | 5.6(5.4-5.8) | 1.4(1.2-1.6) | 4230 | 9.1(8.8-9.3) | | 1.5(1.4-1.7) | 6063 | | 13.0(12.6-13.3) | 1.4(1.3-1.6) |
|  | 2009 | 2840 | | 6.1(5.9-6.3) | 1.5(1.3-1.7) | 4764 | 10.2(9.9-10.5) | | 1.7(1.5-1.9) | 6603 | | 14.1(13.8-14.5) | 1.5(1.4-1.7) |
|  | 2010 | 3014 | | 6.5(6.3-6.7) | 1.6(1.4-1.8) | 5063 | 10.9(10.6-11.2) | | 1.8(1.6-2.0) | 7079 | | 15.2(14.9-15.6) | 1.6(1.5-1.8) |
|  | 2011 | 3150 | | 6.8(6.6-7.0) | 1.6(1.5-1.8) | 5206 | 11.2(10.9-11.5) | | 1.8(1.6-2.0) | 7284 | | 15.7(15.3-16.1) | 1.7(1.5-1.9) |
|  | 2012 | 3463 | | 7.4(7.2-7.7) | 1.8(1.6-2.0) | 5804 | 12.5(12.2-12.8) | | 2.0(1.8-2.3) | 8051 | | 17.3(16.9-17.7) | 1.9(1.7-2.0) |
|  | 2013 | 3228 | | 6.9(6.7-7.2) | 1.7(1.5-1.9) | 5651 | 12.2(11.8-12.5) | | 2.0(1.8-2.2) | 7762 | | 16.7(16.3-17.1) | 1.8(1.6-2.0) |
|  | 2014 | 3457 | | 7.4(7.2-7.7) | 1.8(1.6-2.0) | 5991 | 12.8(12.5-13.2) | | 2.1(1.9-2.3) | 8149 | | 17.4(17.1-17.8) | 1.9(1.7-2.1) |
|  | 2015 | 3663 | | 7.8(7.5-8.0) | 1.9(1.7-2.1) | 6004 | 12.8(12.4-13.1) | | 2.1(1.9-2.3) | 8340 | | 17.7(17.4-18.1) | 1.9(1.7-2.1) |
|  | 2016 | 3574 | | 7.6(7.3-7.8) | 1.8(1.6-2.0) | 6314 | 13.4(13.1-13.7) | | 2.2(2.0-2.4) | 8615 | | 18.3(17.9-18.7) | 1.9(1.8-2.2) |
|  | 2017 | 3581 | | 7.5(7.3-7.8) | 1.8(1.6-2.0) | 6274 | 13.2(12.9-13.5) | | 2.2(1.9-2.4) | 8589 | | 18.1(17.7-18.5) | 1.9(1.7-2.1) |
|  | 2018 | 3186 | | 6.8(6.6-7.0) | 1.6(1.5-1.8) | 5918 | 12.6(12.3-12.9) | | 2.1(1.8-2.3) | 8064 | | 17.2(16.8-17.6) | 1.8(1.7-2.0) |
|  | 2019 | 2895 | | 6.1(5.9-6.4) | 1.5(1.3-1.6) | 5853 | 12.4(12.1-12.7) | | 2.0(1.8-2.3) | 7865 | | 16.6(16.3-17.0) | 1.8(1.6-2.0) |
|  | 2020^d^ | 1699 | | 5.0(4.7-5.2) | 1.2(1.1-1.4) | 2799 | 8.2(7.9-8.5) | | 1.4(1.3-1.6) | 4042 | | 11.8(11.4-12.1) | 1.3(1.2-1.5) |
| Gender | Male | 24266 | | 6.1(6.0-6.1) | Reference =0 | 43068 | 10.7(10.6-10.9) | | Reference =0.043 | 59344 | | 14.8(14.7-14.9) | Reference (p=0.775) |
|  | Female | 24423 | | 6.4(6.3-6.5) | 1.1(1.0-1.1) | 39861 | 10.5(10.4-10.6) | | 1.0(0.9-1.0) | 56195 | | 14.8(14.7-14.9) | 1.0(1.0-1.0) |
| Age group | 15-17 years | 4492 | | 3.9(3.7-4.0) | Reference (p<.001) | 8677 | 7.5(7.3-7.6) | | Reference (p<.001) | 12119 | | 10.4(10.2-10.6) | Reference (p<.001) |
|  | 10-14 years | 10518 | | 4.7(4.6-4.8) | 1.2(1.1-1.3) | 17608 | 7.9(7.8-8.0) | | 1.1(1.0-1.1) | 24652 | | 11.0(10.9-11.2) | 1.1(1.0-1.1) |
|  | 5-9 years | 12200 | | 5.6(5.5-5.8) | 1.4(1.3-1.6) | 16766 | 7.8(7.6-7.9) | | 1.0(1.0-1.1) | 24913 | | 11.5(11.4-11.7) | 1.1(1.0-1.1) |
|  | 1-4 years | 12867 | | 7.8(7.7-8.0) | 1.9(1.7-2.1) | 20981 | 12.8(12.6-13.0) | | 1.6(1.5-1.7) | 29454 | | 17.9(17.7-18.1) | 1.6(1.5-1.7) |
|  | <1 years | 8612 | | 14.2(13.9-14.5) | 3.2(2.9-3.5) | 18897 | 31.2(30.8-31.7) | | 3.5(3.3-3.6) | 24401 | | 40.3(39.8-40.8) | 3.2(3.0-3.4) |
| Deprivation | Least deprived | 2652 | | 1.9(1.8-1.9) | Reference (p<.001) | 7354 | 5.1(5.0-5.3) | | Reference (p<.001) | 9293 | | 6.5(6.4-6.6) | Reference (p<.001) |
|  | 2 | 4267 | | 3.5(3.4-3.6) | 1.9(1.7-2.0) | 8823 | 7.3(7.1-7.4) | | 1.4(1.3-1.4) | 11837 | | 9.7(9.6-9.9) | 1.5(1.4-1.5) |
|  | 3 | 6377 | | 4.5(4.4-4.6) | 2.4(2.2-2.6) | 11609 | 8.2(8.1-8.4) | | 1.6(1.5-1.6) | 15847 | | 11.2(11.1-11.4) | 1.7(1.6-1.8) |
|  | 4 | 10376 | | 6.7(6.6-6.9) | 3.5(3.3-3.8) | 16697 | 10.8(10.7-11.0) | | 2.0(2.0-2.1) | 23566 | | 15.3(15.1-15.5) | 2.3(2.2-2.4) |
|  | Most deprived | 20078 | | 11.1(11.0-11.3) | 5.8(5.4-6.2) | 27443 | 15.2(15.0-15.4) | | 2.8(2.7-2.9) | 40750 | | 22.6(22.4-22.8) | 3.3(3.2-3.5) |
|  | Unknown | 4939 | | 12.1(11.7-12.4) | 4.0(3.6-4.4) | 11003 | 26.9(26.4-27.4) | | 2.7(2.5-2.8) | 14246 | | 34.8(34.2-35.4) | 2.9(2.7-3.1) |
| 1. Event refers to an individual presenting with CM/Possible CM within a given year independent whether there are previous events recorded 2. Adjusted for calendar year, sex, age and deprivation 3. Based on Wald test 4. Data collection to 10.10.2022; denominators adjusted | | | | | | | | | | | | | |

Supplementary Table 2 Hospital admissions, prevalence^a^ per 1000 PYAR (95% CI), IRR^b^(95% CI)^c^ of confirmed CM and possible CM by year, sex, age group and deprivation quintile

|  |  | Confirmed CM | | | Possible CM | | | Confirmed and Possible CM | | |
| --- | --- | --- | --- | --- | --- | --- | --- | --- | --- | --- |
| Variable |  | Events | Incidence  (95% CI) | IRR (95% CI) | Events | Incidence  (95% CI) | IRR  (95% CI) | Events | Incidence  (95% CI) | IRR (95% CI) |
| Year | 2004 | 32 | 0.1(0.0-0.1) | Reference (p<.001) | 1240 | 2.7(2.6-2.9) | Reference (p<.001) | 1265 | 2.8(2.6-2.9) | Reference (p<.001) |
|  | 2005 | 41 | 0.1(0.1-0.1) | 1.2(0.8-1.9) | 1339 | 2.9(2.7-3.1) | 1.1(0.9-1.2) | 1371 | 3.0(2.8-3.1) | 1.1(0.9-1.2) |
|  | 2006 | 60 | 0.1(0.1-0.2) | 1.8(1.2-2.7) | 1448 | 3.1(3.0-3.3) | 1.1(1.0-1.3) | 1483 | 3.2(3.0-3.4) | 1.1(1.0-1.3) |
|  | 2007 | 57 | 0.1(0.1-0.2) | 1.7(1.1-2.5) | 1457 | 3.1(3.0-3.3) | 1.2(1.0-1.3) | 1494 | 3.2(3.1-3.4) | 1.2(1.0-1.3) |
|  | 2008 | 72 | 0.2(0.1-0.2) | 2.1(1.4-3.1) | 1448 | 3.1(2.9-3.3) | 1.1(1.0-1.3) | 1490 | 3.2(3.0-3.4) | 1.1(1.0-1.3) |
|  | 2009 | 79 | 0.2(0.1-0.2) | 2.3(1.5-3.5) | 1445 | 3.1(2.9-3.3) | 1.1(1.0-1.3) | 1502 | 3.2(3.1-3.4) | 1.2(1.0-1.3) |
|  | 2010 | 80 | 0.2(0.1-0.2) | 2.3(1.6-3.4) | 1296 | 2.8(2.6-2.9) | 1.0(0.9-1.2) | 1351 | 2.9(2.8-3.1) | 1.1(0.9-1.2) |
|  | 2011 | 59 | 0.1(0.1-0.2) | 1.7(1.2-2.5) | 1230 | 2.7(2.5-2.8) | 1.0(0.9-1.1) | 1270 | 2.7(2.6-2.9) | 1.0(0.9-1.1) |
|  | 2012 | 40 | 0.1(0.1-0.1) | 1.1(0.8-1.7) | 1066 | 2.3(2.2-2.4) | 0.9(0.8-1.0) | 1096 | 2.4(2.2-2.5) | 0.9(0.8-1.0) |
|  | 2013 | 40 | 0.1(0.1-0.1) | 1.2(0.7-1.8) | 1144 | 2.5(2.3-2.6) | 0.9(0.8-1.0) | 1171 | 2.5(2.4-2.7) | 0.9(0.8-1.0) |
|  | 2014 | 31 | 0.1(0.0-0.1) | 0.9(0.5-1.5) | 1206 | 2.6(2.4-2.7) | 1.0(0.9-1.1) | 1229 | 2.6(2.5-2.8) | 1.0(0.9-1.1) |
|  | 2015 | 25 | 0.1(0.0-0.1) | 0.7(0.4-1.2) | 1133 | 2.4(2.3-2.6) | 0.9(0.8-1.0) | 1154 | 2.5(2.3-2.6) | 0.9(0.8-1.0) |
|  | 2016 | 47 | 0.1(0.1-0.1) | 1.3(0.9-2.0) | 1147 | 2.4(2.3-2.6) | 0.9(0.8-1.0) | 1178 | 2.5(2.4-2.6) | 0.9(0.8-1.0) |
|  | 2017 | 32 | 0.1(0.0-0.1) | 0.9(0.6-1.5) | 1177 | 2.5(2.3-2.6) | 0.9(0.8-1.1) | 1201 | 2.5(2.4-2.7) | 0.9(0.8-1.1) |
|  | 2018 | 37 | 0.1(0.1-0.1) | 1.1(0.7-1.7) | 1009 | 2.2(2.0-2.3) | 0.8(0.7-0.9) | 1041 | 2.2(2.1-2.4) | 0.8(0.7-0.9) |
|  | 2019 | 47 | 0.1(0.1-0.1) | 1.3(0.9-2.0) | 1103 | 2.3(2.2-2.5) | 0.9(0.8-1.0) | 1141 | 2.4(2.3-2.6) | 0.9(0.8-1.0) |
|  | 2020^d^ | 22 | 0.1(0.0-0.1) | 0.9(0.5-1.6) | 487 | 1.4(1.3-1.6) | 0.5(0.5-0.6) | 501 | 1.5(1.3-1.6) | 0.5(0.5-0.6) |
| Gender | Male | 416 | 0.1(0.1-0.1) | Reference (p=0.731) | 13225 | 3.3(3.2-3.4) | Reference (p<.001) | 13507 | 3.4(3.3-3.4) | Reference (p<.001) |
|  | Female | 385 | 0.1(0.1-0.1) | 1.0(0.8-1.1) | 7150 | 1.9(1.8-1.9) | 0.6(0.5-0.6) | 7431 | 2.0(1.9-2.0) | 0.6(0.6-0.6) |
| Age group | 15-17 years | 70 | 0.1(0.0-0.1) | Reference (p<.001) | 1795 | 3.0(2.8-3.1) | 1.0(0.0-0.0) | 1929 | 3.2(3.0-3.3) | 1.0(0.0-0.0) |
|  | 10-14 years | 184 | 0.1(0.1-0.1) | 1.4(1.0-1.8) | 3181 | 1.9(1.9-2.0) | 0.7(0.6-0.7) | 3327 | 2.0(2.0-2.1) | 0.7(0.6-0.7) |
|  | 5-9 years | 134 | 0.1(0.1-0.1) | 1.0(0.8-1.4) | 4124 | 1.9(1.9-2.0) | 0.4(0.4-0.5) | 4218 | 2.0(1.9-2.0) | 0.5(0.4-0.5) |
|  | 1-4 years | 200 | 0.1(0.1-0.1) | 2.0(1.5-2.7) | 6269 | 2.8(2.7-2.9) | 0.5(0.4-0.5) | 6402 | 2.9(2.8-2.9) | 0.5(0.4-0.5) |
|  | <1 years | 213 | 0.4(0.3-0.4) | 5.2(3.9-7.0) | 5006 | 4.3(4.2-4.4) | 0.6(0.5-0.6) | 5062 | 4.3(4.2-4.5) | 0.6(0.6-0.7) |
| Deprivation | Least deprived | 45 | 0.0(0.0-0.0) | Reference (p<.001) | 3045 | 2.1(2.1-2.2) | Reference (p<.001) | 3081 | 2.2(2.1-2.2) | Reference (p<.001) |
|  | 2 | 70 | 0.1(0.0-0.1) | 1.8(1.2-2.7) | 2872 | 2.4(2.3-2.5) | 1.1(1.0-1.2) | 2925 | 2.4(2.3-2.5) | 1.1(1.0-1.2) |
|  | 3 | 82 | 0.1(0.0-0.1) | 1.8(1.2-2.7) | 3413 | 2.4(2.3-2.5) | 1.1(1.1-1.2) | 3466 | 2.5(2.4-2.5) | 1.2(1.1-1.2) |
|  | 4 | 156 | 0.1(0.1-0.1) | 3.1(2.2-4.5) | 4168 | 2.7(2.6-2.8) | 1.3(1.2-1.4) | 4285 | 2.8(2.7-2.9) | 1.3(1.2-1.4) |
|  | Most deprived | 344 | 0.2(0.2-0.2) | 5.9(4.2-8.2) | 5405 | 3.0(2.9-3.1) | 1.4(1.3-1.6) | 5647 | 3.1(3.1-3.2) | 1.5(1.4-1.6) |
|  | Unknown | 104 | 0.3(0.2-0.3) | 3.5(2.4-5.2) | 1472 | 3.6(3.4-3.8) | 1.8(1.6-2.0) | 1534 | 3.7(3.6-3.9) | 1.8(1.6-2.0) |
| 1. Event refers to an individual presenting with CM/Possible CM within a given year independent whether there are previous events recorded 2. Adjusted for calendar year, sex, age and deprivation 3. Based on Wald test 4. Data collection to 10.10.2022; denominators adjusted | | | | | | | | | | |
